# Medical data transformation using rewriting

**DOI:** 10.3389/fninf.2015.00001

**Published:** 2015-02-20

**Authors:** Naveen Ashish, Arthur W. Toga

**Affiliations:** Laboratory of Neuroimaging, Institute for Neuroimaging and Neuroinformatics, Keck School of Medicine of USC, University of Southern CaliforniaLos Angeles, CA, USA

**Keywords:** data integration, Alzheimer's disease datasets, data mapping, query rewriting

## Abstract

This paper presents a system for declaratively transforming medical subjects' data into a common data model representation. Our work is part of the “GAAIN” project on Alzheimer's disease data federation across multiple data providers. We present a general purpose data transformation system that we have developed by leveraging the existing state-of-the-art in data integration and query rewriting. In this work we have further extended the current technology with new formalisms that facilitate expressing a broader range of data transformation tasks, plus new execution methodologies to ensure efficient data transformation for disease datasets.

## Introduction

We present a data transformation system for automatically transforming biomedical data from a data source into a common data model. Our work is part of the GAAIN project (GAAIN, 2014) on creating a federation of multiple Alzheimer's data partners, for integrated access to their data. Our overall approach, which is representative of an entire class of biomedical data federation approaches, is based on the notion of a common data model (Gardner et al., 2001) used to represent the data in any dataset from any data partner. One of the requirements in this approach is that each dataset from each data partner has to be actually transformed to this common data model. This data transformation is the most time consuming and effort intensive phase in integrating any new dataset. Alleviating this effort forms the motivation for our work. The majority of existing data transformation technologies have so far focused on what is the “extract-transform-load” (ETL) model where data transformations, from a dataset to the common model, are specified manually by developers. We provide a declarative approach where data transformations can be specified through logical data transformation rules. The declarative approach offers the advantage of being able to transform new datasets faster as developers have to primarily provide correct data transformation specifications for the new dataset, as opposed to writing custom code for every new data transformation.

Our work is the context of the Global Alzheimer's Association Interactive Network (GAAIN) which is a collaborative project that will provide researchers around the globe with access to a vast repository of Alzheimer's disease research data and the sophisticated analytical tools and computational power needed to work with that data. The goal of GAAIN is to transform the way scientists work together to answer key questions related to understanding the causes, diagnosis, treatment and prevention of Alzheimer's and other neurodegenerative diseases. Medical institutions, universities and centers around the globe collect, synthesize and maintain datasets describing Alzheimer's disease. A variety of data is maintained about each individual subject including basic patient information such as demographic information, overall and specific health and medical characteristics, medication histories, various neurological assessments often as answers to specific and standardized assessment questionnaires, lab data, and also data synthesized from images such as MRI scans or PET scans of the subjects. Such data is often stored in databases, though it may also be present in other storage and analysis software such as SAS (Garcia-Molina et al., 2009), proprietary data management software, or even as files. Seamless access of multiple such datasets from different institutions around the globe holds tremendous value for investigators in this domain, and is the primary motivation for the GAAIN project overall. It is only with the ability to tap multiple disease datasets that investigators can actually find specific cohorts that are large enough for their studies to hold statistical significance. For instance subject characteristics such as “*all subjects with MMSE scores greater than 24 and age above 70yo*” can significantly reduce the size of the cohort as illustrated in the adjoining graphic.

**Figure:**
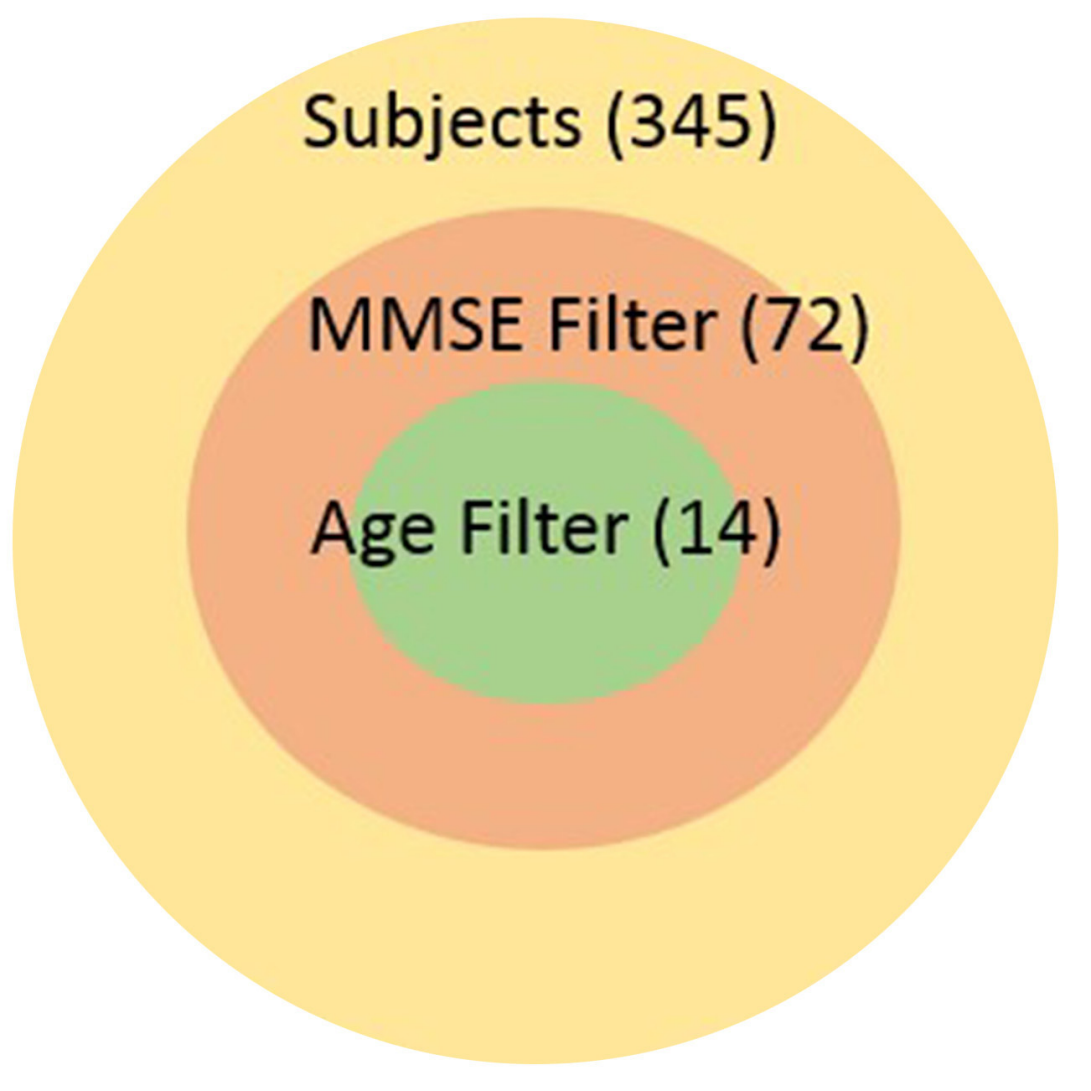


The data transformation system described in this paper has been developed for transforming (Alzheimer's disease) datasets that are to be integrated into the GAAIN network.

## Materials and methods

In this paper we describe our work in the context of two specific datasets that we have transformed and integrated into GAAIN. ADNI, the Alzheimer's Disease Neuroimaging Initiative (Mueller et al., 2008) is a dataset assembled and maintained by the Laboratory of Neuroimaging or LONI (2014). Since 2005, ADNI has been validating the use of biomarkers including blood tests, tests of cerebrospinal fluid, and MRI/PET imaging for Alzheimer's disease (AD) clinical trials and diagnosis. ADNI is maintained as a relational database. The National Alzheimer's Coordinating Center (NACC) was established by the National Institute on Aging in 1999 to facilitate collaborative research. Using data collected from the 29 NIA-funded Alzheimer's Disease Centers (ADCs) across the United States, NACC has developed and maintains a large relational database of standardized clinical and neuropathological research data (Beekly et al., 2007). In partnership with the Alzheimer's Disease Genetics Consortium (ADGC) and the National Cell Repository for Alzheimer's Disease (NCRAD). NACC, like ADNI, provides a valuable resource for both exploratory and explanatory Alzheimer's disease research.

**Common Data Model:** The GAAIN common data model is the integration glue across disparate datasets. This model is currently under development. The common model is expressed as a relational database schema and Table 1 illustrates a subset of the tables in this schema. As an example the GAAIN.SUBJECT table is about basic subject demographics, the GAAIN.HEALTH table has information related to the subjects' diagnosis category (such as whether normal, has AD etc.).

**Table 1:** **GAAIN common data model**.

GAAIN.SUBJECT(SUBJECT_ID,AGE,SEX,RACE,ETHNIC,COUNTRY)
GAAIN.HEALTH(SUBJECT_ID,DIAGNOSIS_M0,DIAGNOSIS_M6,….
DIAGNOSIS_M48, APOE)
GAAIN.COGNITIVE(SUBJECT_ID,MMSE_M0,MMSE_M6, ….,CDR_MO,CDR_M6, …….)

The suffixes “_M0,” “M6” etc., denotes the visit of the subject i.e., the initial visit, the visit after 6 months etc. Our transformation task is to get any relevant dataset that contains all or some of the information for the common model, into the common model schema in Table 1.

**Terminology:** We now formally define some of the technical terms used in this paper. A *federated (database) system* is a type of meta-database management system (DBMS), which transparently maps multiple autonomous database systems into a single virtual database. The *GAAIN federation* is a federation of multiple, distributed database systems or other data sources related to Alzheimer's data. A (GAAIN) *data partner* is an organization that is a member of the GAAIN data federation and provides data to the federation. Any collection of data provided by a data partner is referred to as a dataset. A data partner may have, and provide, multiple datasets. The (GAAIN) *common data model* is a (relational) data model defining common data elements that will be employed in integrating multiple disparate datasets in the GAAIN federation.

**Figure 1:**
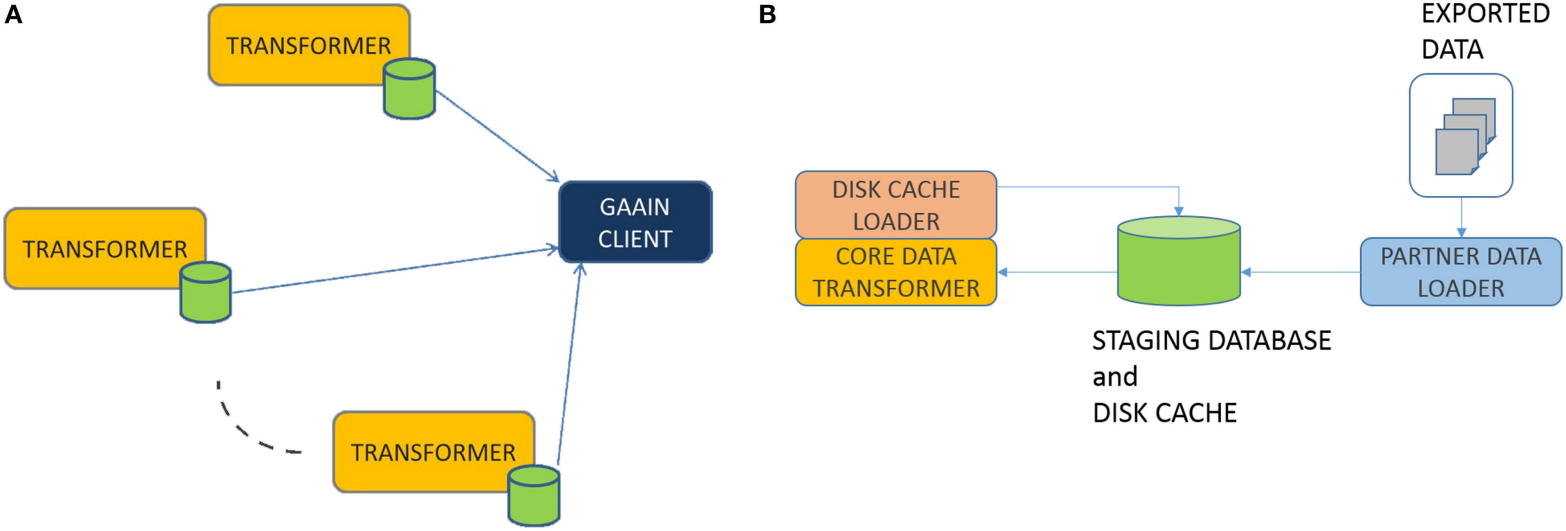
**(A)** Data Transformation. **(B)** Transformer.

**Federation Model:** The overall data federation approach in GAAIN is basically a “map and cache data at data partner site” model as shown in Figure 1A. Any dataset provided by a data partner is transformed into the GAAIN common data model and stored in what is called a (data partner) disk cache, which is a database containing the transformed data. GAAIN clients and applications access data from a data partner by accessing the data in its associated disk cache at run time. Any data transformation software as well as the disk cache itself all reside completely at the data partner site. Such a model has been adopted from the perspective of giving data partners more control over the data they choose to provide into the GAAIN federation. Every data partner site has the Data Transformer software that maps their data into the common model disk cache. We have developed two different approaches to the Data Transformer. One is an approach where the transformer is custom developed for each data transformation task. The data transformations are specified using a graphical data transformation specification tool. The second is a declarative approach where the data transformation system is configured with logical rules that specify the data transformations. This paper describes the second i.e., declarative transformation approach. In the next section we present the technical details of this data transformer.

### Transformer

In this section we describe the box labeled “TRANSFORMER” in Figure 1A above. Overall the process works as follows. Data partners typically provide their data as data exports from their databases or other storage systems. The exported data is typically in the form of a file folder of export files in common formats such as Excel or CSV. The transformer first loads this exported data into a staging database. The staging database is then transformed into the common model disk cache database, by the PARTNER DATA LOADER module. This staging database is a relational database and is what the transformation system actually transforms data from. The CORE DATA TRANSFORMER (Figure 1B) module does the actual data transformation, from the staging database representation to the common model. This is in two phases. In the *Data Elements Transformation* phase we transform individual data elements to their intended representation in the common model. In the *Data Elements Assembly* phase, we assemble transformed individual data elements into the precise data organization i.e., schema required by the common model. Finally, the DISK CACHE LOADER module creates and loads the disk cache with the transformed data. We now describe these modules in more detail.

#### Partner data loader: creating the staging database

The format and protocol for the exported data can vary from partner to partner. We have thus far encountered a few common formats and protocols. For instance, a common format is to have each database table from the source database as a separate spreadsheet and with each spreadsheet containing the data for a table row wise, another format is to have all the source database data in single spreadsheet. The Partner Data Loader handles such formats and loads them into tables in the staging database, and it must be extended in case a new export format is encountered. An important aspect is that while the exported data format itself may vary the organization of the staging database is uniform across any dataset! Regardless of the exported data format, every exported data is loaded into staging database tables of the form.

<key><element>

Table 2 provides an example for 3 data elements from ADNI—RACE, ETHNIC and MMSE where the specified key for the ADNI dataset is the pair (SUBJECT_ID, VCODE). Here SUBJECT_ID is a (unique) subject identifier and VCODE represents the visit code (number) of a particular subject. Each data element is stored in its own separate table along with the key. The primary advantage of this representation is that it serves to make the data transformation rules, discussed below, congruent across different datasets. We will validate this point later in this section.

**Table 2:** **Uniform staging representation**.

STAGING_ADNI_PATIENT_RACE(SUBJECT_ID,VCODE,RACE)
STAGING_ADNI_PATIENT_ETHNIC(SUBJECT_ID,VCODE,ETHNIC)
STAGING_ADNI_PATIENT_MMSE(SUBJECT_ID,VCODE,MMSE)

#### Core data transformer

We distinguish the core data transformation into the two phases of (1) individual data element transformation and (2) data assembly.

***Data elements transformation***. Data elements transformation is transformation at the level of individual elements, or groups thereof. Based upon our domain and datasets, we have characterized the required data transformations into the following five categories: (0) *As-is* The simplest transformation is no transformation at all. As an example the element “MMSE” in the ADNI dataset appears as “MMSE” in the common model as well i.e., there is a common model table with a column named “MMSE” and the source dataset and common model elements correspond to each other in exact terms. (1) *Rename Element Name* In many cases an element in the source dataset appears as a column with a different name in the common model. For instance the element “CDGLOBAL” in the ADNI dataset corresponds to a column called “CDR” in the common model. Actual data values however do not change! (2) *One-to-one Mapping* This is a very common type of mapping and a common scenario this occurs in is if elements are coded differently in the source data vs. the common model. For instance we may represent GENDER as “1” for males, “2” for “females” and 9 for “unknown” in a source dataset, whereas the common model represents these values as “M,” “F,” and “U” respectively. (3) *Many-to-one Mappings* Many to one mappings may also occur, though they are less common. As an example consider the DIAGNOSIS element in the common model which takes values {AD, MCI, NL} depending on whether the subject has Alzheimer's, has memory loss, or is normal, respectively. A data source on the other hand happens to represent this same information in a binary manner, with three separate elements HAS_ALZHEIMERS, HAS_MEMORY_LOSS and IS_NORMAL, each taking a value of “Yes” or “No.” In this case the three elements in the source model must be mapped to the single DIAGNOSIS element in the common model. (4) *Functional Transformations* Finally, functional transformations are those that involve actually computing the data element value from the source data for transformation. Functional transformations may be one to one, or many to one. An example is if we have AGE (in years) as a common model element and the source data provides only a YOB (Year of Birth) element that has the subject's year of birth. In such a case the subject age must be computed from the source data element of YOB. Note that a transformation may fall into multiple categories, for instance one may have a transformation that involves both a renaming of the element name as well as a mapping of the actual data value.

***Data element transformation using rewriting***. In building our data transformation system we leveraged data integration technology for query rewriting. Query rewriting (Beeri et al., 1997) is an extensively explored area in data integration technology (Pinto et al., 2013). The goal of any data integration system is to allow end users and clients to query disparate data sources, in possibly different data models and schemas, using a common model and query language. Query rewriting algorithms and systems provide this capability, and developers are first required to formally state the common model, the content of each data source(s), and most importantly the relationship between the common model and the data elements in each data source. Users address their queries to the common model and the query rewriting system translates the query into the query language of the particular data source(s). In the last two decades a few different formalisms have been employed for the model, source and relationship representation. The most common are (1) Data modeling based on *description logics* and relationship expression using logic based rules (Ives et al., 2012), and (2) Relational modeling, using views to express relationships (Ives et al., 2012).

In our system we have employed a fully implemented query rewriting and mediator system (Ashish et al., 2010) which is based on description logics for data modeling, and *Datalog* style logical rules for specifying data transformations. In Table 3 we explain the formalism using a simple example. Table 3 illustrates what is called a “domain model” for this rewriting system, and it comprises of the following three parts:

**Table 3:** **Domain model**.

**Source_Schema:**	***Common Model Query***
ADNI_RACE(SUBJECT_ID,VCODE,RACE)	SELECT SUBJECT_ID, SEX FROM
ADNI_ETHNIC(SUBJECT_ID,VCODE,ETHNIC)	GAAIN.SUBJECT
ADNI_GENDER(SUBJECT_ID,VCODE,GENDER)	***Rewritten Query***
**Domain_Schema:**	SELECT SUBJECT, PTGENDER as “M”
GAAIN.SUBJECT(SUBJECT_ID,AGE,SEX,RACE,ETHNIC,COUNTRY)	FROM ADNI_PTGENDER
**GAV_Rules:**	WHERE PTGENDER=1
GAAIN.SUBJECT(SUBJECT_ID,“M,”) 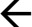	UNION
ADNI_GENDER(SUBJECT_ID,VCODE,GENDER) ^∧^ (GENDER=1)	SELECT SUBJECT, PTGENDER as “F”
GAAIN.SUBJECT(SUBJECT_ID,“F,”) 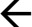	FROM ADNI_PTGENDER
ADNI_GENDER(SUBJECT_ID,VCODE,GENDER)^∧^ (GENDER=2)	WHERE PTGENDER=2
GAAIN.SUBJECT(SUBJECT_ID,“U,”) 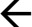	UNION
ADNI_GENDER(SUBJECT_ID,VCODE,GENDER)^∧^ (GENDER=9)	SELECT SUBJECT, PTGENDER as “U”
	FROM ADNI_PTGENDER
	WHERE PTGENDER=9

**Source Schema:** The source schema is the part of the domain model that describes the contents in the data source that we are transforming data *from*, in this case the staging database. In the example in Table 3 (left column) the part under “Source_Schema:” describes the schema of the ADNI source data, in this case three of the staging data tables. A complete domain model would contain such a description for *all* the tables in the staging database. **Domain Schema** This part describes the schema of the data that we are transforming *to*, which is the common data model. The part under “Domain_Schema:” shows one of the common data model tables, for subject demographic information. A complete domain model would contain such a description for all the tables in the common data model. **GAV Rules** “GAV” stands for “global-as-view” (Ashish et al., 2010) which should be thought of as a formalism where a data transformation is specified as describing domain schema data elements in terms of source schema data elements. This part is the actual transformation logic and, as mentioned earlier, the rule language is logic based. The first rule states the following “*The element SEX in the table GAAIN.SUBJECT corresponds to the element GENDER in the ADNI data source table ADNI_GENDER. It will take a value of M for all subjects when the source data has a value of 1*.” The following two rules are also essentially the same structurally, except that they encode the mappings of dataset values “2” (to “F”) and “9” (to “U”) respectively.

The domain model must thus contain the descriptions of all database tables in the staging database to be mapped, all tables in the common model, and finally sound and complete transformation rules for each data element that is to be mapped. Domain model specification is the most time consuming and effort intensive part for transforming any new dataset. Once a domain model has been correctly and completely specified however, we have the required machinery for actual data transformation. The rewriting system is now capable of transforming any valid query to the common model, to a query that is (entirely) in terms of the underlying data source. Table 3 (right column) provides a simple example where we have a common model query in terms of the GAAIN.SUBJECT common model table. This query is rewritten to the query illustrated below it, the rewritten query is exclusively in terms of the tables in the underlying staging database. The transformation of data elements from the staging database to the common model is thus powered completely by such rewritten queries where we can now load data elements in the common model tables using corresponding rewritten queries on the staging database tables.

***Data assembly***. Data transformation rules provide a means for transforming individual data elements. However collections of elements must be assembled meaningfully together into the exact schema specified in the common model. While it may appear that the data elements transformation is the major part of the entire data transformation task, the transformed data assembly phase poses significant complexity. Consider the following common model table:

GAAIN.ASSESSMENT (SUBJECT_ID, MMSE_M0, MMSE_M6, MMSE_M12, …, MMSE_M24, MMSE_M36, MMSE_M48)

The data elements MMSE_MO, MMSE_M6 through MMSE_M48 refer to the “MMSE” score values of a subject. The suffix _M0 denotes the score at the subject's initial visit, _M6 denotes the score at the 6 months after or second visit, etc.

Table 4 shows two possible kinds of transformation rules we may write for this example. Unfortunately neither of these two sets of rules will accomplish the desired transformation. In the first set of rules we will not be able to capture any subjects who do not have values (of the element MMSE score) for all seven of their visits! The second set of rules will end up creating *separate* tuples for a single subject, one for each of their visits. This limitation is due to the fact that one can express database join operations in such transformation rules, but not *outer-joins* (Garcia-Molina et al., 2009) which is what is required here. Further, the more significant issue here is that the rewritten query in this case would result in a 7-way join (on the ADNI_MMSE staging table). For a staging table of a reasonable size of say 100,000 records, and even with the database table indexed appropriately, this join is inefficient. Finally, for the above example one has to write this kind of a rule seven times over, which is inefficient. We now present new formalisms with accompanying execution strategies to address these issues.

**Table 4:** **Transformation rules**.

**GAAIN.COGNITIVE**(SUBJECT_ID,MMSE_M0,MMSE_M6,MMSE_M12,MMSE_M18,MMSE_M24,MMSE_M36,MMSE_M48) 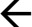
**ADNI_MMSE**(SUBJECT_ID,VISIT,MMSE_M0) ^∧^ (VISIT=1)
^∧^
…
**ADNI_MMSE**(SUBJECT_ID,VISIT,MMSE_M48) ^∧^ (VISIT=7)
**GAAIN. COGNITIVE**(SUBJECT_ID,MMSE_M0,,) 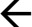 **ADNI_MMSE**(SUBJECT_ID,VISIT,MMSE_M0) ^∧^ (VISIT=1)
…
**GAAIN. COGNITIVE**(SUBJECT_ID,,MMSE_M48) 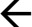 **ADNI_MMSE**(SUBJECT_ID,VISIT,MMSE_M48) ^∧^ (VISIT=7)

**Iterative Rule:** We introduce a new kind of data transformation rule, called the iterative rule. An iterative rule is nothing but succinct notation for representing what actually is a set of multiple rules with the same structure. Formally, an iterative rule is of the form:

(<key>,<element>)[<iterator>:<range>|<set of element names>]

<conjunctive rule body> ^∧^ <conjunctive rule constraints with iterator>

Here:

<key> is a set of elements representing the identifying key.

<element> is the data element to be transformed.

<iterator> is an element

<range> represents the set of values for the <iterator> element, it may be specified as a discrete set of value or as a numerical (integer) range

<set of element names> is the set of names that the target element will take when the iteration is expanded

Table 5 (left column) provides an example of an iterative rule. In the above example the <key> is the set of elements {SUBJECT_ID}, the <element> is MMSE, the <iterator> is “i,” its range is 1 to 7, and the set of element names is {MMSE_M0, …,MMSE_48}. The conjunctive rule body is the first predicate and the iterator constraints is the second predicate—which is (VISIT=i). The right column in Table 5 shows the actual (multiple) rules that this iterative rule gets expanded to. We simply create a rule for each possible value of the iterator i, from 1 to 7 in this case.

**Table 5:** **Iterative rules**.

**(SUBJECT_ID,MMSE)**[i:1-7|	**MMSE**(MMSE_M0) 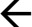 **ADNI_MMSE**(SUBJECT_ID,VISIT,MMSE) ^∧^ (VISIT=1)
MMSE_M0,MMSE_M6,MMSE_M12,MMSE_M18,	**MMSE**(MMSE_M6) 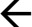 **ADNI_MMSE**(SUBJECT_ID,VISIT,MMSE) ^∧^ (VISIT=2)
MMSE_M24,MMSE_M36,MMSE_M48]	**…**
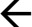	**MMSE**(MMSE_M48) 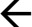 **ADNI_MMSE**(SUBJECT_ID,VISIT,MMSE) ^∧^ (VISIT=7)
** ADNI_MMSE**(SUBJECT_ID,VISIT,MMSE) ^∧^ (VISIT=i)	

The second primitive we introduce relates to the execution strategy for such rules. A more efficient alternative to the JOIN operation is the SQL MERGE operation. MERGE is an operator (Garcia-Molina et al., 2009) to add new records or update existing ones (when conditions match). It was introduced in in the SQL:2003 standard (Garcia-Molina et al., 2009). This new primitive enables specifying an entire *collection* of data transformations at one time, which will ultimately be assembled (collectively) using the SQL MERGE operation. Our second new primitive thus is the *RuleSet* A RuleSet is a specification that comprises of the following 3 elements: (a) Target Table: A target table, is a (single) table in the common data model. (b) Key: A key is a set of columns that forms a primary key for the target table. (c) Rules: Transformation rules.

A rule set captures the logic to query multiple data elements, all indexed by the same key, and load them into a target table indexed by that key. Table 6 (left column) provides an example of a rule set. Any iterative rules are first expanded to actual instantiated rules. Each rule provides the transformation for a single data element i.e., we can query that element in terms of the common model and obtain a corresponding rewritten query to the staging database. Each element is finally inserted into the common model database using a MERGE statement, where the MERGE operation is into the specified (common model) target database table and the actual data comes from the rewritten staging database query. Table 6 (right column) illustrates the final MERGE statements resulting this from the rule set example. The specific steps to generate the final transformation execution script, given a rule set specification are shown in Table 7.

**Table 6:** **Rule sets**.

**Target table**: GAAIN.COGNITIVE	MERGE INTO GAAIN.ASSESSMENT(SUBJECT_ID,MMSE_M0)
**Key**: SUBJECT_ID	KEY(SUBJECT_ID)
**Rules**:	SELECT SUBJECT_ID,MMSE from ADNI_MMSE WHERE VISIT=1
**(SUBJECT_ID,MMSE)**[i:1-7|	….
MMSE_M0,MMSE_M6,MMSE_M12,MMSE_M18,	MERGE INTO GAAIN.ASSESSMENT(SUBJECT_ID,MMSE_M48)
MMSE_M24,MMSE_M36,MMSE_M48]	KEY(SUBJECT_ID)
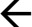	SELECT SUBJECT_ID,MMSE from ADNI_MMSE WHERE VISIT=7
**ADNI_MMSE**(SUBJECT_ID,VISIT,MMSE) ^∧^ (VISIT=i)	

**Table 7:** **Transformation script**.

**Given:**
RuleSet RS=<T,K,RS>
**Return:**
Transformation SQL Script
**Procedure:**
for each rule r ε R
ER 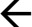 expand (r)
M 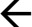 generateDomainModel(ER)
rewriter 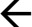 instantiateDomainModel(M)
for each rule r ε ER
H 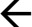 getHead(r)
V 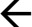 getElement(r)
RWQ 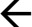 rewrite(select ^*^ from”+H)
sqlQ 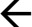 MERGE INTO
TABLE+T+VALUES(K,V), key(K)+RWQ
finalScript 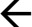 add(sqlQ)
return finalScript

### Disk cache loader: creating and populating the disk cache

The final step is to create and populate the disk cache database. This is simply a set of the following tasks: (1) Create the GAAIN common model schema, in a local database, with a provided common model script. (2) Execute the final script obtained above. The final script will access the staging database during execution.

**Rule Congruence:** In Table 8 we provide an example with two transformation rules for two different datasets, ADNI and NACC. As the example shows, the corresponding rules for either of the elements SEX or RACE, are structurally identical across ADNI and NACC. The only difference is in (1) The elements used as keys in ADNI vs. NACC, and (2) The element names on the staging database side i.e., ADNI refers to SEX as GENDER whereas NACC refers to it as SEX, ADNI refers to RACE as RACE and NACC refers to it as PATRACE. This kind of rule congruence makes writing new data transformation rules for additional datasets much easier, as transformation rules written for prior datasets can be modified with relatively less effort to new datasets.

**Table 8:** **Congruent rules**.

**ADNI rules**	**NACC rules**
G.SEX(SUB_ID,SEX) 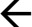 ADNI_GENDER (SUB_ID,VISIT,SEX)	G.SEX(SUB_ID,SEX) 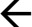 NACC_SEX(NDC,SUB_ID,VISIT,SEX)
G.RACE(SUB_ID,RACE) 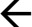 ADNI_RACE(SUB_ID,VISIT,RACE)	G.RACE(SUB_ID,RACE) 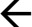 NACC_PATRACE(NDC,SUB_ID,VISIT,RACE)

While such a “one table per element” staging representation can lead to a very large number of staging tables, in practice the common model typically has a relatively small number of data elements. Thus the set of elements that has to be loaded into the staging database is a small subset of all elements in the dataset and the staging database remains tractable to maintain and query. To address applications where a larger number of source data elements need to be in the staging database (for instance in the common model has a larger number of elements), we are investigating (i) A single table representation of all required source data elements in the staging database, and (ii) A NoSQL framework (Sadalage and Fowler, 2013) for the representing staging database, that is likely more scalable.

## Results

Our experimental evaluation is in the context of data transformation for the GAAIN project. We have empirically evaluated two specific aspects, (1) The effort and time required to integrate new data partners and their datasets into the federation, (2) Performance, in terms of the time taken for transforming a given dataset. For the time and effort assessment recall that we have introduced two capabilities to make this task easier which are (i) A uniform key-element organization in the staging database to ensure transformation rule congruence across datasets and (ii) The introduction of Iterative Rule and RuleSet formalisms to be able to write rules more succinctly. We evaluated the time required to develop a sound and complete set of transformation rules based on the time required by developers to actually do the task. We recruited a set of 12 medical informatics students and programmers with familiarity with data management technologies and some familiarity with data integration, which is representative of the kind of personnel that have to configure medical data integration systems in practice. The developers were also provided with technical documentation on transformation rules, as well as some documentation of the data in Alzheimer's datasets. We divided the developers into two groups, one who would use only existing transformation rule formalisms and the staging database created as-is and the other who would indeed use the new formalisms where applicable and the uniform staging database.

As Table 9 shows, the new rule formalisms provided about a 25% reduction in rule writing effort. The uniform staging plus congruent rulesets provided a *near 75% reduction* in effort for writing the transformation rules for NACC, once the rules for ADNI had already been written.

**Table 9:** **Effort optimization**.

**Dataset**	**ADNI**	**NACC**
**STRATEGY**		
Baseline	21 h	20 h
Iterative rules	16 h	17 h
Uniform staging + congruent rules	15 h	5 h

**Figure 2:**
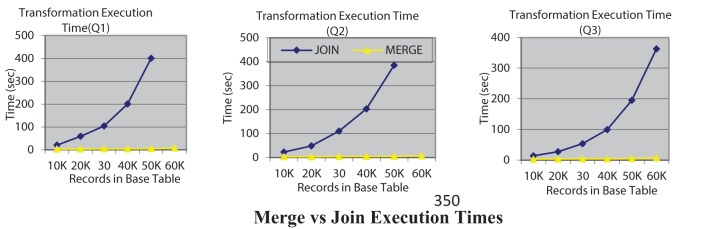
**Merge vs. join execution times**.

Our second experiment is to evaluate and compare the data transformation execution time of join-based execution of rewritten queries with the new merge-based execution that we have introduced. Figure 2 shows the query execution times for both options, JOIN and MERGE for three queries where the rewritten query can be executed using either option. The execution times clearly indicate the utility of the MERGE based execution. In fact for queries Q1 and Q2 with the join option the query execution for 60 K records did not terminate even in an hour. All experiments were conducted on a Dell Precision T5500 server with an Intel Xeon 2.67 GHz processor, running Windows 7 with 12 GB RAM. It must be mentioned that while the algorithmic complexity of the MERGE and JOIN alternatives is the *same*—it is *O(N logN)* in both the MERGE and JOIN options where N is the number of record in a table, it is the implementation of the MERGE operation that is significantly more efficient in many database systems that is leading to this improved efficiency.

## Discussion

The general area of data integration in life sciences, especially based on a data federation, has seen many contributions in the last 10 years. Many of the efforts such as (Louie et al., 2007; Azami et al., 2012) have focused on a mediated approach to data integration. In recent years the idea of integrating disparate health or biomedical datasets based on a common data model has gained ground. Some examples include the SCANNER project (Nguyen et al., 2012) on clinical data integration using the OMOP model (Ogunyemi et al., 2013), the PEN effort on exercise datasets harmonization, the NDAR national data repository (Hall et al., 2012) for autism research and the Fontan integrated data repository. The work in (Detwiler et al., 2009) focuses on an XQuery driven mediation based approach to Alzheimer's data integration but which is restricted to small groups of collaborators and also groups that are willing to share their data externally within the group—a significant distinction from the GAAIN model. The DISCO (Marenco et al., 2014) framework is focused on data integration in support of the “NIF” (Marenco et al., 2014) portal and the integration is at the level of data aggregation as opposed to actual data fusion that GAAIN aims for. The OpenfMRI (Poldrack et al., 2013) project also takes a shared data repository paradigm where the assumption is that different groups would indeed convert their data to a shared common model and also provide it to a shared data repository for integration. There is also a significant body of work in ontology driven approaches to biomedical data integration (Bodenreider, 2008), which is required when semantic knowledge resources, like particular biomedical ontologies, need to be integrated (for search and retrieval purposes) as well. In the GAAIN domain the datasets are almost exclusively relational and thus a relational common model suffices to represent the data from any dataset. Even with a well-defined common data model for integration, there remains the challenge of converting data from each provider into the common model. The majority of the current data transformation efforts in the medical informatics domain employ ETL based tools for manually transforming their data. Our system is, to the best of our knowledge, a first effort toward a complete declarative transformation approach. One contribution of our work is leveraging and adapting existing data integration technology specifically for query rewriting to the data elements transformation aspect of data transformation. A second contribution is the extension of rule language for data assembly, accompanied with the introduction of an efficient merge based execution that achieves an efficient execution of the data assembly phase in the data transformation. Finally, we have introduced the uniform key-value representation scheme for the staging database in any data transformation task, and demonstrated how that leads to rule congruence across different datasets and transformation tasks and ultimately to reduced effort in developing transformation rules for new datasets.

The GAAIN data transformer is currently employed for actual transformation of Alzheimer's datasets in the GAAIN project. While this paper has focused on the core transformation system, there are several related research issues that we are currently actively working on. The first is the problem of schema matching. At present the mapping of elements of a dataset to the common model is manual which is time consuming and tedious. While approaches have been developed for automatic or semi-automatic schema matching and mapping (Madhavan et al., 2005) they largely rely on the availability of mapping training data already available. We are working on a schema mapping approach that leverages information in data dictionaries available from any data partner. A second problem is the integrity of data transformations. Our approach is to apply techniques from formal program verification to data transformation. From the spectrum of available techniques, verification via theorem proving is well suited for this task given that data transformations are specified using formal logic. Finally, we are also investigating enhancing staging database scalability with frameworks such as NoSQL.

### Conflict of interest statement

The authors declare that the research was conducted in the absence of any commercial or financial relationships that could be construed as a potential conflict of interest.
